# Supplementary material for: Co-dependent and Interdigitated: Dual Quorum Sensing Systems Regulate Conjugative Transfer of the Ti Plasmid and the At Megaplasmid in Agrobacterium tumefaciens 15955
Source: Front Microbiol. 2021 Jan 18;11:605896. doi: 10.3389/fmicb.2020.605896 (PMC7856919; doi:10.3389/fmicb.2020.605896)
Supplement: Supplementary file 1 [file Data_Sheet_1.pdf]

SUPPLEMENTARY INFORMATION

Co-Dependent and Interdigitated: Dual Quorum Sensing Systems Regulate Conjugative Transfer of the Ti Plasmid and the At Megaplasmid in *Agrobacterium tumefaciens* 15955

Ian S. Barton, Justin L. Eagan, Priscila Nieves-Otero, Ian P. Reynolds,
Thomas G. Platt and Clay Fuqua

Supplementary Tables (S1-S3), Supplementary Figure Legends, Supplementary References, Supplementary Figures (S1-S6)

SUPPLEMENTAL TABLES

Table S1: Strains used in this study

Strain	Genotype/markers	Notes	Reference
E. coli			
DH5α/λpir	λpir ; cloning strain		(Chiang and Rubin 2002)
S17-1/λpir	λpir ; Tra^{+} , cloning host		(Kalogeraki and Winans 1997)
A. tumefaciens			
C58-ERM52	Sp^{R}	Plasmidless (pAt-, pTi-) Conjugation recipient	(Morton et al. 2014)
NTL4	C58 Δ*tetRA*	pTi-cured derivative of *A. tumefaciens*, AHL^{-}	(Luo et al. 2001)
15955-KT1	Δ*traI*^{Ti}		This study
15955-KT2	Δ*traI*$^{At\_1}$		This study
15955-KT3	Δ*traI*^{Ti}; Δ*traI*$^{At\_1}$		This study
15955-IB123	pTi15955::*gusA*Gm^{R}		Barton et al. 2019
15955-IB125	pAt15955::Km^{R}		Barton et al. 2019
15955-IB137	pAt15955::Km^{R}; Δ*traI*^{Ti}	KT1, marked on pAt15955 with Km^{R}	This study
15955-IB138	pAt15955::Km^{R}; Δ*traI*$^{At\_1}$	KT2, marked on pAt15955 with Km^{R}	This study
15955-IB139	pAt15955::Km^{R}; Δ*traI*^{Ti}; Δ*traI*$^{At\_1}$	KT3, marked on pAt15955 with Km^{R}	This study
15955-IB148	pTi15955::*gusA*Gm^{R}; Δ*traI*^{Ti}	KT1, marked on pTi15955 with *gusA* and Gm^{R}	This study
15955-IB149	pTi15955::*gusA*Gm^{R}; Δ*traI*$^{At\_1}$	KT2, marked on pTi15955 with *gusA* and Gm^{R}	This study
15955-IB150	pTi15955::*gusA*Gm^{R}; Δ*traI*^{Ti}; Δ*traI*$^{At\_1}$	KT3, marked on pTi15955 with *gusA* and Gm^{R}	This study
15955-PAG1	Δ*traR*^{Ti}		This study
15955-IAM1	Δ*traR*^{At}		This study
15955-PAG9	Δ*traR*^{Ti}Δ*traR*^{At}		This study

Table S2: Plasmids used in this study

Plasmid	Features	Reference
pIB302	pSRKGm P_{lac}-*traI*$^{At\_1}$	This study
pIB303	pSRKGm P_{lac}-*traI*^{Ti}	This study
pIB305	pSRKGm P_{lac}-*traI*$^{At\_2}$	This study
pIB306	pSRKGm P_{lac}-*traR*^{At}	This study
pIB307	pSRKGm P_{lac}-*traR*^{Ti}	This study
pIB308	pSRKKm P_{lac}-*traR*^{At}	This study
pIB309	pSRKKm P_{lac}-*traR*^{Ti}	This study
pIB310	pRA301 P_{traA}^{At}-*lacZ*	This study
pIB311	pRA301 P_{traI}^{At}-*lacZ*	This study
pIB312	pRA301 P_{traA}^{Ti}-*lacZ*	This study
pIB313	pRA301 P_{traI}^{Ti}-*lacZ*	This study

Table S3: Primers used in this study*

Table S3: Primers used in this study*

Primer	Sequence	Description
IBP156	aggcatatgttggagcggac	pAt15955 *traI*_2 (truncated) 5' w/NdeI for expression in pSRK
IBP157	aggactagttcatccatctttgc	pAt15955 *traI*_2 (truncated) 3' w/SpeI for expression in pSRK
IBP158	aggcatatgcgaatcttgac	pAt15955 *traI*_2 (modified) 5' w/NdeI for expression in pSRK
IBP159	ggaactagttcatccatctttgc	pAt15955 *traI*_2 (modified) 3' w/SpeI for expression in pSRK
IBP160	ggacatatgcaacattggct	pAt15955 *traR* 5' w/NdeI for expression in pSRK
IBP161	aggactagttcagatcagcccgg	pAt15955 *traR* 3' w/SpeI for expression in pSRK
IBP162	ggacatatgcagcactggct	pTi15955 *traR* 5' w/NdeI for expression in pSRK
IBP163	aggactagttcagatgagtttcc	pTi15955 *traR* 3' w/SpeI for expression in pSRK

IBP190	aggcatatgcggatcctgaccgttcc	pAt15955 *traI*_1 5' w/NdeI for expression in pSRK
IBP191	aggactagttcacgccgcgctcctcgccg	pAt15955 *traI*_1 3' w/SpeI for expression in pSRK
IBP192	aggcatatgctgattctgaccgtctc	pTi15955 *traI* 5' w/NdeI for expression in pSRK
IBP193	aggactagttcacgccgcactcctcaacg	pTi15955 *traI* 3' w/SpeI for expression in pSRK
IBP210	gaattcgagctcggtaccttttgcgcgcatctgatgtcatcg	pRA301 (SphI/KpnI) *lacZ* fusion, *traA*^{pAt} 5’
IBP212	gaattcgagctcggtaccagcgttttgtgcgaagtgg	pRA301 (SphI/KpnI) *lacZ* fusion, *traI*^{pTi} 5’
IBP213	ggagcaagcttgcatgccatgttgatctccgcaacaagaaacga	pRA301 (SphI/KpnI) *lacZ* fusion, *traI*^{pTi} 3’
IBP216	gaattcgagctcggtaccgctcgctaccggtccggct	pRA301 (SphI/KpnI) *lacZ* fusion, *traA*^{pTi} 5’
IBP217	ggagcaagcttgcatgccacggcgaagtgcgctcccgg	pRA301 (SphI/KpnI) *lacZ* fusion, *traA*^{pTi} 3’
IBP218	ggagcaagcttgcatgccacggcgaagagcgctcccgg	pRA301 (SphI/KpnI) *lacZ* fusion, *traA*^{pAt} 3’
IBP219	gaattcgagctcggtaccccgatttcgccttgatcggg	pRA301 (SphI/KpnI) *lacZ* fusion, *traI*^{pAt} 5’
IBP220	ggagcaagcttgcatgccatatctttctccgctt	pRA301 (SphI/KpnI) *lacZ* fusion, *traI*^{pAt} 3’

Note:

*All other primers used in strain creation or diagnostics are available upon request.

SUPPLEMENTARY FIGURE LEGENDS

Figure S1. Comparative gene organization within pAt and pTi from *A. tumefaciens* 15955 and *A. tumefaciens* C58. (A) Whole replicon gene cluster locations of pAt15955, pAtC58, pTi15955, and pTiC58 from *A. tumefaciens* 15955 and *A. tumefaciens* C58 (also known as *A. fabrum*). Light blue bar labeled pAt15955Δ270 indicates the segment precisely deleted upon curing of pTi15955 previously described (Barton et al. 2019) **(B)** Comparison of *traM-traR* gene neighborhoods for pAt15955, pTi15955 and pTiC58. Gene names and unannotated gene numbers provided from NCBI annotation of the *A. tumefaciens* 15955 and *A. tumefaciens* C58 genome sequences. **(C)** Comparison of *traI* gene neighborhoods for pAt15955, pTi15955 and pTiC58. $TraI^{At\_2}$ is included in crosshatching and with a solid outline, but was found to not produce a detectable AHL. Solid black vertical lines on gene maps mark locations of *tra* box elements.

Figure S2. Clustal omega alignment of TraI sequences from pTi15955 and pAt15955. Conserved residues within the AHL synthase family are highlighted in red (Churchill and Herman 2008).

Figure S3. Conjugation of pAt15955 is restored in *A. tumefaciens* 15955 Δ$traI^{Ti}$ Δ$traI^{At_1}$ upon exogenous addition of 3-oxo-C8-HSL. *A. tumefaciens* 15955 Δ$traI^{Ti}$ Δ$traI^{At_1}$ harboring a plasmid-borne copy of P_{lac}-*traR* from either pAt15955 (blue) or pTi15955 (green) were mixed 1:1 with a plasmidless recipient (ERM52) and spotted

onto ATGN media containing 400 μM IPTG and varying amounts of 3-oxo-C8-HSL (X axis). After 24 hours, conjugation frequencies (Y axis) were calculated as transconjugants per output donor (see Materials and Methods).

Figure S4. Clustal omega alignment of TraR sequences from pTi15955 and pAt15955. Conserved residues within the AHL coordination and DNA-binding domains are highlighted in orange or red, respectively. Arrow indicates single amino acid difference within the DNA-binding domain of pAt and pTi TraR, and asterisks mark residues R/K206 and R/E210 in each protein (White and Winans 2007).

Figure S5. Ectopic expression of either *traR* gene in *traR* null mutants increases expression of *traI* targets. *A. tumefaciens* 15955 and mutant derivatives (Δ*traR*^{Ti}, Δ*traR*^{At}, and Δ*traR*^{Ti}Δ*traR*^{At}) carrying a plasmid-borne copy of *traR* either from pTi15955 (solid bars) or from pAt15955 (striped bars) and *traI-lacZ* fusions from either pTi15955 (green) or pAt15955 (blue) plasmid were spotted on 0.2 μm cellulose acetate filter discs placed on ATGN alone (light fill) or with 400 μM IPTG (dark fill) to induce expression of the *traR* gene, and incubated at 28°C for 48 h. Cells were resuspended and promoter activities were calculated as Miller Units (Methods). Bars are standard deviation. All bars that differ by 10-fold or more are significant (p-value <0.05). Bars that differ by less than 10-fold, that are however significantly different from the corresponding wild type background (p-value <0.05) are designated with an asterisk or double asterisk.

Figure S6. Alignment of subset of putative *tra* boxes from pAt and pTi in *A. tumefaciens* 15955 and motif analysis. (A) Inverted repeats of putative *tra* box elements are indicated by black arrows. Approximate -35 and -10 elements and +1 sites are indicated by gray boxes or bolded, respectively. Bolded residues are putative transcriptional start sites. (B) Putative *tra* boxes from pAt15955 or pTi15955 (Figure S10A) were used to create a sequence motif with WebLogo (Crooks et al. 2004).

SUPPLEMENTARY REFERENCES

Chiang SL, Rubin EJ. 2002. Construction of a mariner-based transposon for epitope-tagging and genomic targeting. Gene.296:7.

Churchill ME, Herman JP. 2008. Acyl-homoserine lactone biosynthesis: structure and mechanism. In: Chemical Communication among Bacteria. Washington, D.C.: ASm Press. p. 275-290.

Crooks GE, Hon G, Chandonia JM, Brenner SE. 2004. WebLogo: a sequence logo generator. Genome Res. Jun;14:1188-1190. Epub 2004/06/03.

Kalogeraki VS, Winans SC. 1997. Suicide plasmids containing promoterless reporter genescan simultaneously disrupt and create fusions to target genes of diverse bacteria. Gene.188:7.

Luo Z-Q, Clemente TE, Farrand SK. 2001. Construction of a Derivative of *Agrobacterium tumefaciens* C58 That Does Not Mutate to Tetracycline Resistance. MPMI.14:6.

Morton ER, Platt TG, Fuqua C, Bever JD. 2014. Non-additive costs and interactions alter the competitive dynamics of co-occurring ecologically distinct plasmids. Proceedings Biological sciences / The Royal Society. Mar 22;281:20132173.

White CE, Winans SC. 2007. Cell-cell communication in the plant pathogen *Agrobacterium tumefaciens*. Philos Trans R Soc Lond B Biol Sci. Jul 29;362:1135-1148. Epub 2007/03/16.

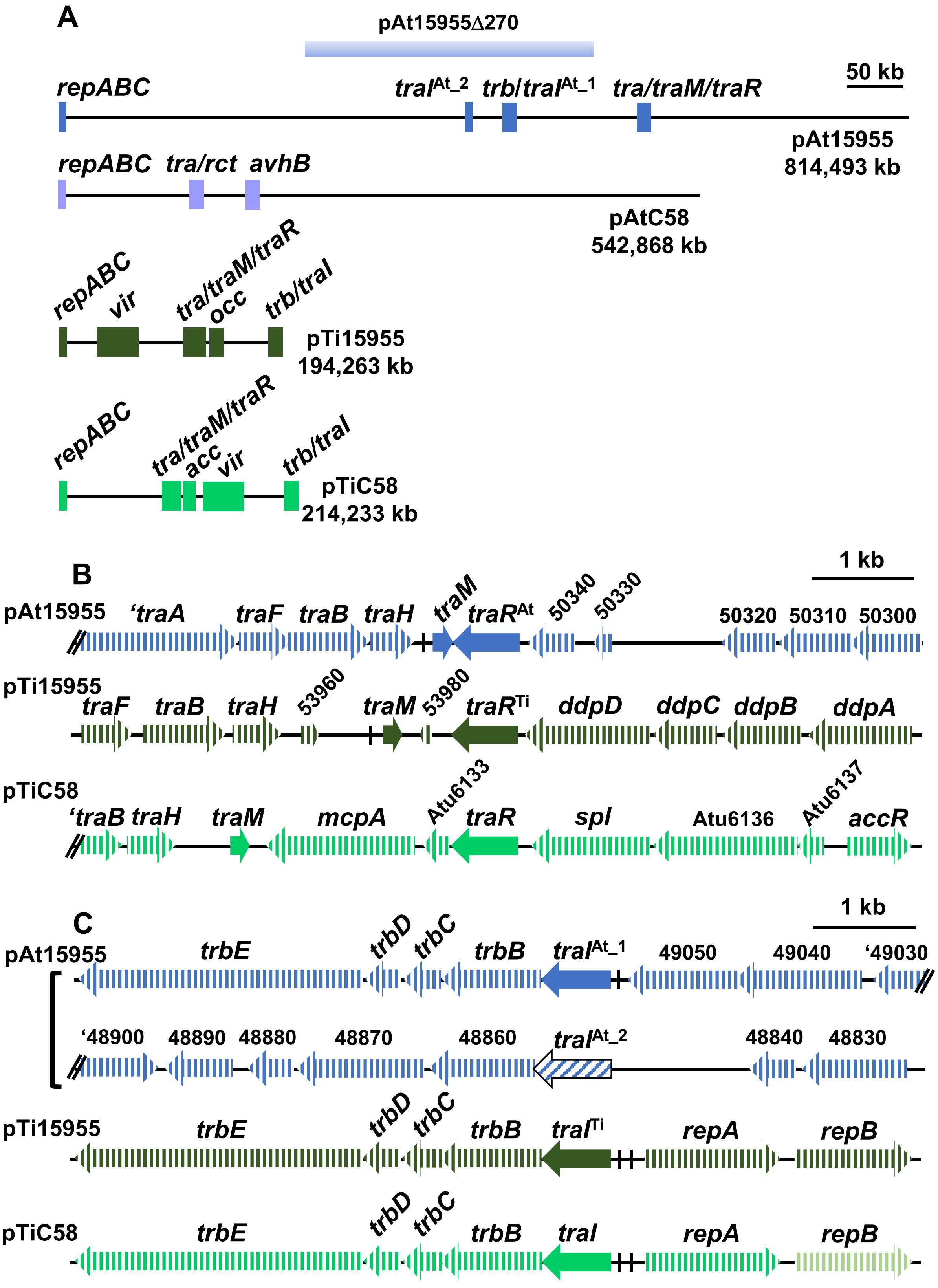

Barton et al.; Figure S1

```
TraI^Ti     MLILTVSPDQYQHQNSYLKQMHRLRAEVFGNRLKWDVAIEDGGERDQYDELSPTYILATF     60
TraI^At_1   MRILTVPPDQHAHHKDELAQMHRLREMVFRGRLEWDVTVTETGEFDEYDQFDPTYVLAVT     60
TraI^At_2   MRILTVPRDQYTDQSDELAQMHRLRAIGFRDRLERGVTTSEGGEFDQRDQLDPTNIRAVA     60
            * ****  **: .:.. * ******   * .**: .*:  : ** *: *::.** : *.

TraI^Ti     GGQRVVGCARLLPASGPTMLERTFPQLLATGSLSATTAMIESSRFCVDTTLPTGRAGRQL     120
TraI^At_1   DYGRVVGCARLLPAVGPTMLELTFPQLLRDGSLNATSAMIESSRFCVDTTLPAGRGGGQL     120
TraI^At_2   NDNRVVVCARRLPAVGRTMLERTFPQLLASGAFNAIYRMIESFRVCIDTSFP-GRGGGQF     119
            .  *** *** *** * **** ******  *::.*   **** *.*:**::* **.* *:

TraI^Ti     HLATLTMFAGIIEWSMANGYDEIVTATDLRFERILKRAGWPMTRLGEPVAIGNTVAVAGH     180
TraI^At_1   HLATLTMFAGIIEWSMANGYDKIVTATDLRFERILNRAGWPMARLGEPVAIDNTVAIAGT     180
TraI^At_2   HLTALTVFAFIIELSMANGYDQIVTAKDLDFECTRNRPRWPITRPGEPVEIDNTIAISGT     179
            **::**:** *** *******:****.** **   :*  **::* **** *.**:*::*

TraI^Ti     LPADRKSFERVCPPGYRSIIADDNGRPLRSAA--------------------------     212
TraI^At_1   LPADQESFEQVRRPNYRSTLSYGDNHSARSAA--------------------------     212
TraI^At_2   LPTDQASFEQVCPMGYRSITSGNGVDPAGAKTSAAVPPRMTNLKARRNSAQVRTCKDG     237
            **:*: ***:*   .***  : ..     : :
```

Barton et al.
Figure S2

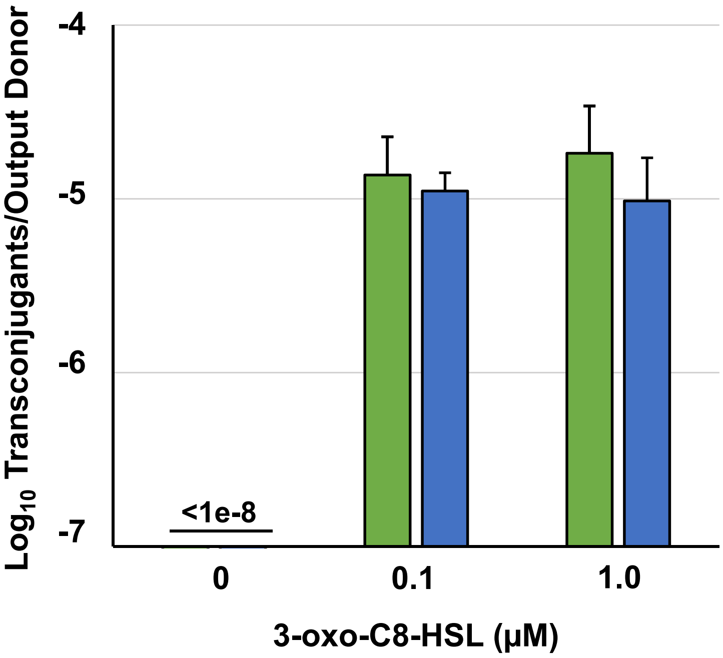

Barton et al.
Figure S3

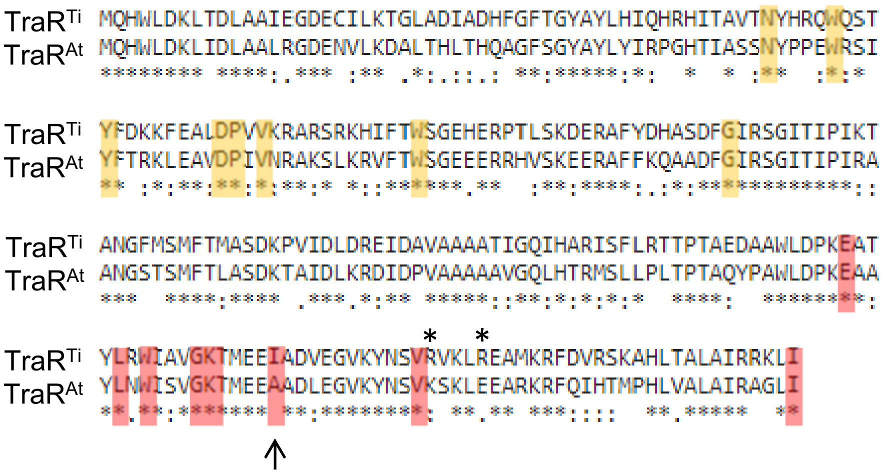

Barton et al.
Figure S4

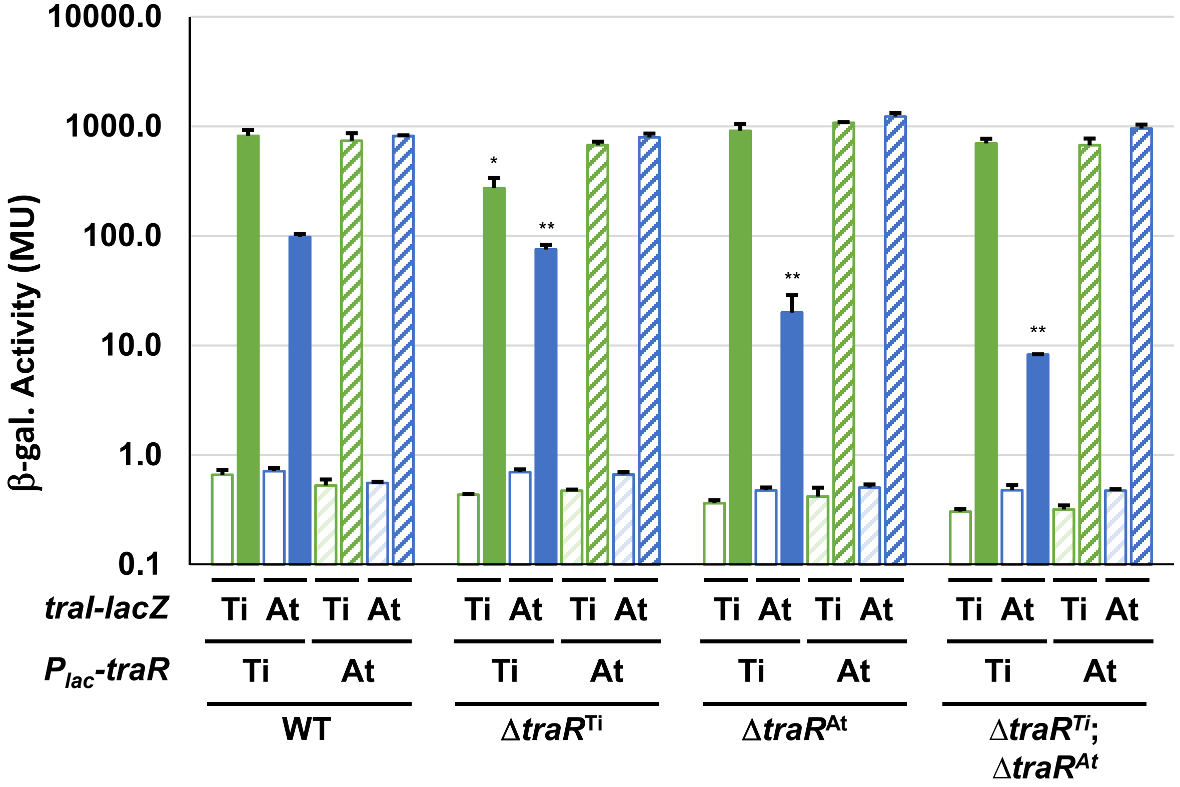

Barton et al.
Figure S5

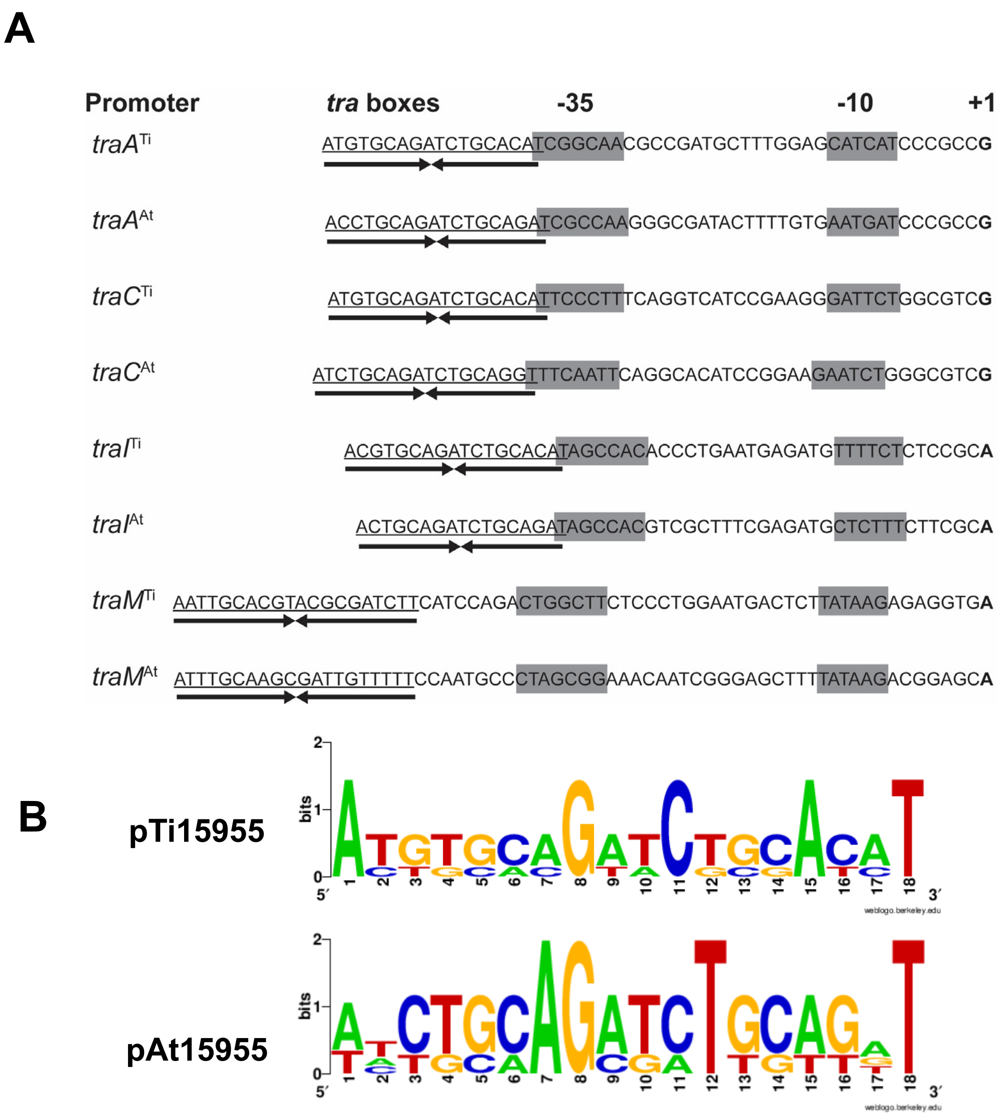

Barton et al.
Figure S6
